# Can we predict patient outcome before extracorporeal membrane oxygenation for refractory cardiac arrest?

**DOI:** 10.1186/s13049-020-00753-6

**Published:** 2020-06-23

**Authors:** Fu-Yuan Siao, Chun-Wen Chiu, Chun-Chieh Chiu, Yu-Jun Chang, Ying-Chen Chen, Yao-Li Chen, Yung-Kun Hsieh, Chu-Chung Chou, Hsu-Hen Yen

**Affiliations:** 1 Department of Emergency Medicine, Changhua Christian Hospital, Changhua, Taiwan; 2 Department of Critical Care Medicine, Changhua Christian Hospital, Changhua, Taiwan; 3 Department of Mechanical Engineering, Chung Yuan Christian University, Taoyuan, Taiwan; 4 Epidemiology and Biostatistics Center, Changhua Christian Hospital, Changhua, Taiwan; 5 Department of Cardiovascular Surgery, Changhua Christian Hospital, Changhua, Taiwan; 6 Department of Internal Medicine, Changhua Christian Hospital, Changhua, Taiwan; 7 College of Medicine, Chung-Shan Medical University, Taichung, Taiwan

**Keywords:** Refractory cardiac arrest, Out-of-hospital cardiac arrest, In-hospital cardiac arrest, Emergency department cardiac arrest, Extracorporeal cardiopulmonary resuscitation, Extracorporeal membrane oxygenation

## Abstract

**Background:**

Refractory cardiac arrest resistant to conventional cardiopulmonary resuscitation (C-CPR) has a poor outcome. Although previous reports showed that extracorporeal cardiopulmonary resuscitation (E-CPR) can improve the clinical outcome, there are no clinically applicable predictors of patient outcome that can be used prior to the implementation of E-CPR. We aimed to evaluate the use of clinical factors in patients with refractory cardiac arrest undergoing E-CPR to predict patient outcome in our institution.

**Methods:**

This is a single-center retrospective study. We report 112 patients presenting with refractory cardiac arrest resistant to C-CPR between January 2012 and November 2017. All patients received E-CPR for continued life support when a cardiogenic etiology was presumed. Clinical factors associated with patient outcome were analyzed. Significant pre-ECMO clinical factors were extracted to build a patient outcome risk prediction model.

**Results:**

The overall survival rate at discharge was 40.2, and 30.4% of patients were discharged with good neurologic function. The six-month survival rate after hospital discharge was 36.6, and 25.9% of patients had good neurologic function 6 months after discharge. We stratified the patients into low-risk (*n* = 38), medium-risk (*n* = 47), and high-risk groups (*n* = 27) according to the TLR score (low-flow **T**ime, cardiac arrest **L**ocation, and initial cardiac arrest **R**hythm) that we derived from pre-ECMO clinical parameters. Compared with the medium-risk and high-risk groups, the low-risk group had better survival at discharge (65.8% vs. 42.6% vs. 0%, *p* < 0.0001) and at 6 months (60.5% vs. 38.3% vs. 0%, *p* = 0.0001). The low-risk group also had a better neurologic outcome at discharge (50% vs. 31.9% vs. 0%, *p* = 0.0001) and 6 months after discharge (44.7% vs. 25.5% vs. 0%, *p* = 0.0003) than the medium-risk and high-risk groups.

**Conclusions:**

Patients with refractory cardiac arrest receiving E-CPR can be stratified by pre-ECMO clinical factors to predict the clinical outcome. Larger-scale studies are required to validate our observations.

## Background

Extracorporeal cardiopulmonary resuscitation (E-CPR) is being increasingly used as an effective supportive tool for prolonged cardiac arrest refractory to conventional cardiopulmonary resuscitation (C-CPR) [1–3]. E-CPR implementation is a complex procedure that requires extensive teamwork for successful cardiopulmonary life support [4, 5]. The procedure can have major complications, such as massive bleeding and brain injury [6, 7], and it has a high financial burden [4, 8]. Despite increased E-CPR use for prolonged cardiac arrest, the survival rate varies from 8.8% [9] to 54% [10], depending on candidate selection, and the overall survival to discharge is 29% [11].

Recent guidelines from the American Heart Association recommend E-CPR use as a class IIb recommendation to improve survival in patients with potentially reversible refractoriness to C-CPR [12]. Although E-CPR may help bridge patients to diagnosis or therapy, it may also bridge patients to nowhere, raising ethical concerns [13]. Because E-CPR is usually performed in the emergency setting when the patient is unconscious, it is difficult to conduct large-scale studies to compare E-CPR and C-CPR results in the real world [14]. Data regarding appropriate patient selection criteria for E-CPR from randomized comparative trials are lacking [15]. However, observational clinical studies aimed at identifying those who may least benefit from such advanced techniques are needed to prevent futile extracorporeal membrane oxygenation (ECMO) use [16, 17]. For example, the Survival After Veno-arterial ECMO (SAVE) score was developed to predict patient survival following cardiogenic shock using clinical parameters [18, 19], and the ENCOURAGE mortality risk score from a French study group was proposed to predict mortality in ECMO-treated patients with acute myocardial infarction [20]. Laboratory data and etiology of cardiac arrest are often unknown prior to E-CPR use. Using such prognostic scores is complex and requires clinical information that is typically only available after ECMO use. Prognostic factors, such as low-flow time and cardiac rhythm, were found to be related to survival and neurological outcome in patients with in-hospital cardiac arrest (IHCA) or out-of-hospital cardiac arrest (OHCA) who received E-CPR [21–23]. Such prognostic factors have not been combined to predict survival in patients with refractory cardiac arrest receiving E-CPR. In our previous studies, E-CPR was shown to improve patient survival and neurological outcome [3]. In our experience, traditionally poor prognostic factors, such as low-flow time as long as 250 min [1] or nonshockable cardiac rhythm [24], can be appropriately managed with E-CPR to achieve good neurological outcome. In the present study, we aimed to review the clinical outcomes of patients in our institution with refractory cardiac arrest receiving E-CPR and identify potentially useful clinical parameters before ECMO therapy initiation to predict patient outcome in this challenging condition.

## Methods

### Patients

Medical charts between January 2012 and November 2017 in the hospital were retrospectively reviewed. The hospital was the only medical center equipped to perform 24/7 E-CPR service in Changhua County, with an area of 1074 km^2^ and a population of 1.2 million located in central Taiwan. The Institutional Review Board of Changhua Christian Hospital approved the study (IRB No. 141103). We enrolled patients who fulfilled the following criteria for the E-CPR program: age 18–75 years; cardiac arrest presumed to be of cardiac origin; C-CPR initiated for cardiac arrest within 5 min (no-flow time ≤ 5 min); and refractory cardiac arrest defined as failure to achieve return of spontaneous circulation (ROSC) after at least 10 min of C-CPR.

**Fig. 1:**
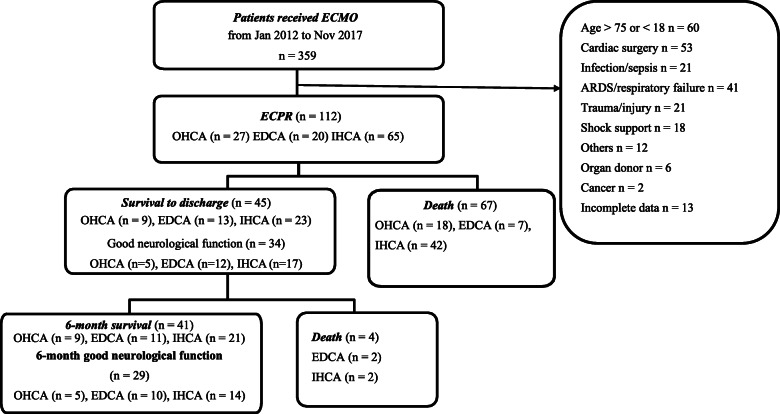
Patient allocation flow chart

Patients were excluded if the time from cardiac arrest onset to C-CPR activation was prolonged or unknown. They were also excluded if they had severe head trauma, acute active bleeding, severe sepsis, terminal cancer, or any history of severe neurological deficits (including dementia, ischemic stroke, intracranial hemorrhage, and bedridden state). In total, 112 patients with refractory cardiac arrest were identified and enrolled in this study (Fig. 1). OHCA was defined as cardiac arrest occurring outside the hospital, and IHCA was defined as cardiac arrest occurring in the hospital during hospital admission. Emergency department cardiac arrest (EDCA) was defined as cardiac arrest occurring in the emergency department. The low-flow time represents the period from initiation of any CPR (either bystander CPR or professional CPR) to initiation of femoral cannulation [3].

### Assessment of resuscitation process and clinical outcome

The number of cardiac arrests, etiology of cardiac arrest, cardiac arrest rhythm, and time from CPR to ECMO were retrospectively reviewed. Sustained ROSC was defined as > 20 min of spontaneous circulation without cardiac arrest recurrence. Neurological outcome was evaluated using the Glasgow–Pittsburgh cerebral performance category (CPC) scale. Good neurological outcome was defined as a CPC score of 1 or 2, poor neurological function as a CPC score of 3 or 4, and brain death as a CPC score of 5. Patients were evaluated for survival and good neurological function at discharge and 6 months after discharge. Information on the patients who survived E-CPR until hospital discharge was collected from medical records and telephone interviews.

### ECMO system and intervention

In our hospital, E-CPR was initiated as an option for prolonged CPR. The attending physician and our ECMO team will discuss the patient’s situation and make the judgment in terms of above-mentioned inclusion criteria. The ECMO system included a centrifugal blood pump, oxygenator, patient outflow cannula, and patient inflow cannula. The pump flow was controlled to maintain a minimum flow of 2.0 L/min. The activated clotting time was maintained at 180–220 s with heparin. We implemented E-CPR via femoral cannulation in the emergency department, regular medical ward, intensive care unit, or cardiac catheterization laboratory. Patients who achieved sustained ROSC after E-CPR were transferred to subsequent intervention for diagnosis and treatment. The patient was declared dead if ROSC was not achieved after 90 min of E-CPR. Emergency coronary angiography was performed for patients with suspected acute coronary syndrome. Targeted temperature management was considered when the patient remained comatose after ROSC and was determined by the attending physician of the intensive care unit.

### Statistical analyses

Demographic data and other clinical data of continuous variables are presented as median and interquartile range (IQR, 25th–75th percentile), whereas categorical variables are presented as number and percentage. Independent variables were first analyzed by univariate methods. The Mann–Whitney U test was used to compare median values of continuous variables between the groups, whereas the chi-square test or Fisher’s exact test was used for categorical variables. Receiver operating characteristic (ROC) curves were used to investigate the time from CPR to ECMO, lactate, and initial pH to identify survivors at discharge. The Youden index was used to determine the optimal cut-off values. Survival analysis was evaluated using the Kaplan–Meier method and log rank test to assess the effect of risk level on the likelihood of death at 6 months after VA ECMO initiation in patients.

Variables that achieved statistical significance (*p* < 0.05) in univariate analysis were subsequently subjected to multivariate analysis using logistic regression analysis. To derive survival component scores, each predictor’s coefficient retained in the multiple logistic regression model was divided by the model’s smallest coefficient, multiplied by 5, and rounded to the nearest integer. The predictive accuracy of this scaled score was quantified with area under the ROC curve (AUC) estimates. Goodness of fit was verified by the Hosmer–Lemeshow test. Internal validation of the risk score was performed using 1000 bootstrap resamples. All statistical analyses were performed using IBM SPSS Statistics for Windows, Version 22.0 (IBM Corp., Armonk, NY) and SAS (version 9.4; SAS Institute, Cary, NC).

## Results

### Patient characteristics and outcome of refractory cardiac arrest with E-CPR

In total, 359 patients received ECMO therapy at our hospital during the study period, of whom 112 patients who met the inclusion criteria were enrolled in the study (Fig. 1). The majority of the patients were male (74.1%; median age, 59.5 years). The causes of cardiac arrest were acute myocardial infarction (58.9%), cardiomyopathy (11.6%), myocarditis (10.7%), pulmonary embolism (8.0%), and others (10.7%). Cardiac arrests were classified by location as IHCA (58.0%), OHCA (24.1%), or EDCA (17.9%). Furthermore, 63 patients (56.2%) presented with initial shockable rhythm, and the remaining patients presented with nonshockable rhythm, including asystole and PEA. The patients had a median low-flow time (CPR to ECMO time) of 46.0 min (IQR, 35.0–57.0 min). Overall, 45 patients (40.2%) survived to discharge, and 41 patients (36.6%) were alive during the 6-month follow-up period. Furthermore, 34 patients (30.4%) had good neurological outcome at discharge, and 29 patients (25.9%) had good neurological outcome during the 6-month follow-up period.

### Comparison of clinical outcome between survivors and nonsurvivors at discharge

Table 1 shows the comparison between survivors and nonsurvivors at discharge. Compared with nonsurvivors, survivors had similar underlying disease, a trend toward younger age (55.0 years [IQR, 45.0–66.0] vs 61.0 years [IQR, 54.0–68.0], *p* = 0.102), a higher proportion of acute myocardial infarctions (*p* = 0.030), a higher proportion of initial shockable rhythms (*p* = 0.0002), and a different distribution of the location of cardiac arrest (*p* = 0.043). Survivors had a significantly shorter low-flow time (CPR to ECMO time) (*p* = 0.001). Compared with nonsurvivors, survivors had lower lactate (*p* = 0.004), higher initial pH (*p* = 0.014), and similar troponin (*p* = 0.350) and creatinine levels (*p* = 0.426). Survival to discharge was higher for EDCA than for OHCA (65.0% vs. 33.3%, *p* = 0.0333) or IHCA (65.0% vs. 35.4%, *p* = 0.0198) and did not differ between OHCA and IHCA (33.3% vs. 35.4%, *p* = 0.8516). The rate of good neurological outcome at discharge was higher for EDCA than for OHCA (60% vs. 18.5%, *p* = 0.0038) or IHCA (60% vs. 26.2%, *p* = 0.0055) and did not differ between OHCA and IHCA (18.5% vs. 26.2%, *p* = 0.4368).

**Table 1:** Demographic characteristics of patients after refractory cardiac arrest

			Survival to discharge		
		Total *N* = 112 (100%)	No *N* = 67 (59.8%)	Yes *N* = 45 (40.2%)	*P*-value
Variable		N (%)	N (%)	N (%)	
Gender	Male	83(74.1)	52(77.6)	31(68.9)	0.302
Age	Median (IQR) ^*a*^	59.5 (50.0–67.5)	61.0 (54.0–68.0)	55.0 (45.0–66.0)	0.102
	≥ 55	72 (64.3)	49(73.1)	23(51.1)	0.017
Etiology of cardiac arrest	Acute myocardial infarction	66 (58.9)	38 (56.7)	28 (62.2)	0.030
	Cardiomyopathy	13 (11.6)	9 (13.4)	4 (8.9)	
	Myocarditis	12 (10.7)	3 (4.5)	9 (20.0)	
	Pulmonary embolism	9 (8.0)	8 (11.9)	1 (2.2)	
	Others	12 (10.7)	9 (13.4)	3 (6.7)	
Underlying disease	Coronary artery disease	100 (89.3)	59 (88.1)	41 (91.1)	0.759
	Hypertension	62 (55.4)	39 (58.2)	23 (51.1)	0.459
	Diabetes mellitus	53 (47.3)	32 (47.8)	21 (46.7)	0.909
	Pulmonary disease	28 (25.0)	17 (25.4)	11 (24.4)	0.911
	Renal insufficiency	36 (32.1)	23 (34.3)	13 (28.9)	0.546
	Chronic liver disease	12 (10.7)	6 (9.0)	6 (13.3)	0.539
	Hyperlipidemia	26 (23.2)	16 (23.9)	10 (22.2)	0.839
Location of cardiac arrest	OHCA^*b*^	27 (24.1)	18 (26.9)	9 (20.0)	0.043
	EDCA^*c*^	20 (17.9)	7 (10.4)	13 (28.9)	
	IHCA^*d*^	65 (58.0)	42 (62.7)	23 (51.1)	
Initial cardiac rhythm	Asystole/PEA	49 (43.8)	39 (58.2)	10 (22.2)	0.002
	Pulseless VT^*e/*^ Vf^*f*^	63 (56.2)	28 (41.8)	35 (77.8)	
CPR to ECMO (min)	Median (IQR)	46.0 (35.0–57.0)	50.0 (42.0–60.0)	38.0 (35.0–46.0)	0.001
	≥ 48	53 (47.3)	43 (64.2)	10 (22.2)	<0.001
Lactate (IU/ml)	Median (IQR)	8.6 (5.7–13.1)	9.8 (6.6–15.3)	7.0 (5.2–9.5)	0.004
Troponin (ng/L)	Median (IQR)	3.2 (0.7–24.4)	3.6 (0.7–37.2)	2.5 (0.5–11.0)	0.350
Initial pH	Median (IQR)	7.3 (7.1–7.3)	7.2 (7.1–7.3)	7.3 (7.2–7.4)	0.014
Creatinine (mg/dL)	Median (IQR)	1.6 (1.2–2.0)	1.7 (1.3–2.0)	1.4 (1.1–1.9)	0.426
PCI	Yes	75 (67.0)	47 (70.1)	28 (62.2)	0.382
IABP	Yes	60 (53.6)	39 (58.2)	21 (46.7)	0.230
Dialysis	Yes	34 (30.4)	19 (28.4)	15 (33.3)	0.575
Therapeutic Hypothermia	Yes	52 (46.4)	31 (46.3)	21 (46.7)	0.967
ROSC	Yes	108 (96.4)	63 (94.0)	45 (100.0)	0.147
ECMO duration (hour)	Median (IQR)	71.0 (26–124)	44.5 (19–101)	93.0 (66–148)	<0.001
Hospital stay (day)	Median (IQR)	14.0 (3.5–27.0)	5.0 (2.0–16.0)	24.0 (16.0–47.0)	<0.001
Follow-up time (month)	Median (IQR)	0.6 (0.1–25.5)	0.1 (0.0–0.5)	33.8 (15.0–48.6)	<0.001
Neurologic function at discharge (CPC score)	1	32 (28.6)	0 (0.0)	32 (71.1)	<0.001
	2	2 (1.8)	0 (0.0)	2 (4.4)	
	3	5 (4.5)	0 (0.0)	5 (11.1)	
	4	6 (5.4)	0 (0.0)	6 (13.3)	
	5	67 (59.8)	0 (0.0)	0 (0.0)	
Good neurologic function at discharge	Yes (CPC ≤ 2) ^*g*^	34 (30.4)	0 (0.0)	34 (75.6)	<0.001
Survival-6 months later	Yes	41 (36.6)	0 (0.0)	41(91.1)	
Neurologic function-6 month later (CPC score)	1	29 (25.9)	0 (0.0)	29 (64.4)	<0.001
	2	0 (0.0)	0 (0.0)	0 (0.0)	
	3	6 (5.4)	0 (0.0)	6 (13.3)	
	4	6 (5.4)	0 (0.0)	6 (13.3)	
	5	4 (3.6)	0 (0.0)	4 (8.9)	
Good neurologic function-6 months later	Yes (CPC ≤ 2)	29 (25.9)	0 (0.0)	29 (64.4)	

^a^*IQR* interquartile range

^b^*OCHA* out-of-hospital cardiac arrest

^c^*EDCA* emergency department cardiac arrest

^d^*IHCA* in-hospital cardiac arrest

^e^*VT* ventricular tachycardiaf

^f^*Vf* ventricular fibrillation

^g^*CPC* cerebral performance category

### Risk score construction and risk stratification of patient outcome after E-CPR

**Fig. 2:**
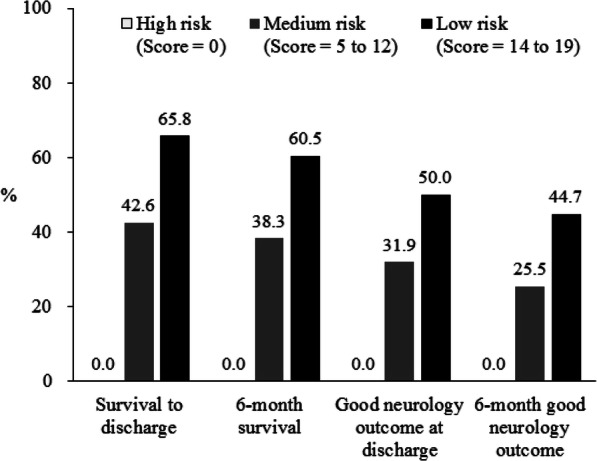
Clinical outcome according to risk group

**Fig. 3:**
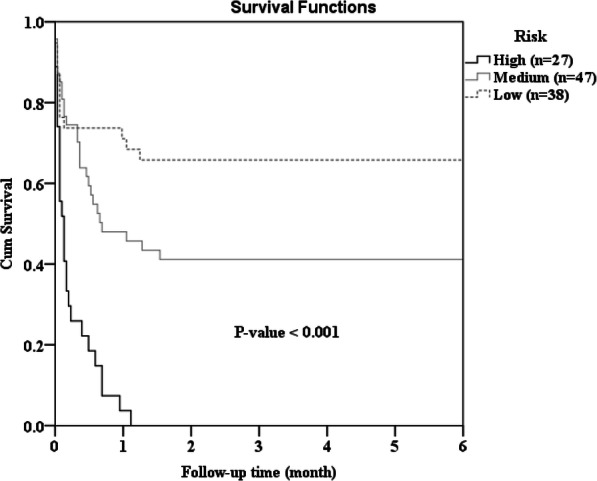
Survival analysis according to risk group. Kaplan–Meier survival curves for extracorporeal cardiopulmonary resuscitation in high-risk, medium-risk, and low-risk groups (*p* < 0.0001)

In the univariate logistic regression analysis, the following variables significantly affected survival to discharge: age, location of cardiac arrest, CPR to ECMO, initial cardiac rhythm, serum lactate concentration, initial pH, and cardiac arrest etiology. To stratify patient outcome before E-CPR use, we extracted clinically important factors before E-CPR use (Table 2) according to univariate and multivariate analyses. Laboratory data (serum lactate concentration and initial pH) and cardiac arrest etiology are often unknown prior to ECMO and were not included in the multivariate analysis. Patient age lost its significance in the multivariate analysis. Three factors (cardiac arrest location, CPR to ECMO time, and initial cardiac rhythm) were identified as significant, with survival component scores of 5, 7, and 7 points, respectively. The survival risk of each patient was divided into three mortality risk levels: score = 0, defined as high; score 5–12, defined as medium; and score 14–19, defined as low. Then, we compared the clinical outcome between the three risk groups. In total, 27 patients (24.1%) were classified as high risk, 38 patients (33.9%) as low risk, and the remaining patients as medium risk (Table 3). There were significant differences in clinical outcome between these three groups. Patients in the low-risk group had the best clinical outcome, with a survival to discharge rate of 65.8% and a survival to 6 months after discharge rate of 60.5%. The rate of good neurological outcome in the low-risk group was 50% at discharge and 44.7% at 6 months after discharge (Fig. 2). The high-risk group had the worst clinical outcome, with none of the patients surviving at discharge (Fig. 3).

**Table 2:** Logistic regression analysis of survival to discharge

	Univariate analysis (crude)			Multivariate analysis (adjusted)			
Predictor	Coefficient	OR(95% CI)	*P*-value	Coefficient	OR(95% CI)	*P*-value	Score
Age							
< 55	0.957	2.604 (1.175–5.771)	0.018				
> =55		1.000					
Location of cardiac arrest							
OHCA^a^ or IHCA^*b*^		1.000			1.000		
Emergency department	1.248	3.482(1.263–9.600)	0.016	1.312	3.715(1.190–11.596)	0.024	5
CPR^*c*^ to ECMO^*d*^ (min)							
< 48	1.836	6.271(2.648–14.851)	< 0.001	1.743	5.714(2.221–14.698)	< 0.001	7
> =48		1.000			1.000		
Initial cardiac rhythm							
Asystole or PEA^*e*^		1.000			1.000		
Pulseless VT^*f*^ or Vf^*g*^	1.584	4.875(2.075–11.453)	< 0.001	1.712	5.543(2.057–14.937)	0.001	7
Lactate (IU/ml)							
< =9.7	1.269	3.556(1.451–8.711)	0.006				
> 9.7		1.000					
Initial pH							
< 7.31		1.000					
> =7.31	1.171	3.225(1.394–7.464)	0.006				
Eitology of cardiac arrest							
Acute myocardial infarction		1.000					
Cardiomyopathy	−0.506	0.603(0.169–2.158	0.437				
Myocarditis	1.404	4.071(1.009–16.426)	0.049				
Pulmonary embolism	−1.774	0.170(0.020–1.435)	0.103				
Others	−0.793	0.452(0.112–1.825)	0.265				

Hosmer-Lemeshow goodness-of-fit test indicated no evidence of lack of fit in the selected model (*p* = 0.185).OR = Odds ratio

^*a*^*OHCA* Out-of-hospital cardiac arrest, ^*b*^*IHCA* In-hospital cardiac arrest, ^*c*^*CPR* Cardiopulmonary resuscitation, ^*d*^*ECMO* Extracorporeal membrane oxygenation, ^*e*^*PEA* Pulselss electrical activity, ^*f*^*VT* Ventricular tachycardia, ^*g*^*Vf* Ventricular fibrillation

**Table 3:** Clinical outcome according to risk stratification of patients

Risk Group		Survival to discharge		Survival 6 months after discharge		Good function at discharge		Good function 6 months after discharge	
	Total	N	%	N	%	N	%	N	%
	112	45	40.2	41	36.6	34	30.4	29	25.9
High (S = 0)	27	0	0.0	0	0.0	0	0.0	0	0.0
Medium (S = 5–12)	47	20	42.6	18	38.3	15	31.9	12	25.5
Low (S = 14–19)	38	25	65.8	23	60.5	19	50.0	17	44.7

## Discussion

In this study, we report that patients with refractory cardiac arrest had an overall survival rate of 40.2, and 30.4% of patients were discharged with good neurological function after E-CPR use. These findings are consistent with those of previous studies of E-CPR for prolonged cardiac arrest [21–23, 25]. We attempted to stratify our patients into three clinical risk groups according to three clinical parameters (cardiac arrest location, low-flow time, and initial cardiac rhythm) before initiating E-CPR. A high rate (65.8%) and a low rate (0%) of survival to discharge can be discriminated with this clinical classification system. This is the first attempt to use pre-ECMO parameters to predict patient outcome in cases of refractory cardiac arrest. This finding may aid in shared decision-making regarding ECMO use when prolonged cardiac massage occurs in daily practice.

The prognosis for patients with prolonged cardiac arrest is ominous when they are refractory to C-CPR [26], with survival rates of less than 1%. E-CPR has the ability to restore blood flow to prevent metabolic dysfunction in the highly lethal situation of prolonged cardiac arrest [26, 27]. Previous investigations have shown that E-CPR can improve the clinical outcome in patients with IHCA beyond 10 min [19, 28, 29]. Observational studies have also found that early E-CPR use may help to improve the neurological outcome in patients with prolonged cardiac arrest [3, 5, 24, 30]. Early studies found a more favorable outcome of E-CPR among IHCA patients than among OHCA patients [7, 22, 25] because of a shorter interval from cardiac arrest to active ECMO support among IHCA patients. A subsequent study showed that the clinical outcome of E-CPR can be similar between IHCA and OHCA patients with appropriate patient selection [31]. One strength of the present study is that we identified a unique type of cardiac arrest, EDCA. In this series with appropriate patient selection, we found no differences in clinical outcome between the IHCA and OHCA groups. Although E-CPR use in emergency departments was limited in a recent survey in the United States [32], we found that the EDCA group had better clinical outcome than the IHCA and OHCA groups. When patients present to the emergency department with subsequent development of cardiac arrest, these patients are more likely to receive prompt and high-quality CPR than other patients [33]. Even with prolonged cardiac arrest, these patients are most likely to have good prognostic factors and deserve aggressive E-CPR initiation in such a situation [3, 4].

The time interval from CPR initiation to ECMO is correlated with patient outcome. Previous studies showed that low-flow time (from CPR to ECMO) was strongly correlated with survival rate and neurological outcome [34, 35]. In the present study, the low-flow time was longer in nonsurvivors than in survivors (50 vs. 38 min, *p* < 0.001). Thus, earlier identification of patients with refractory cardiac arrest who are suited for E-CPR treatment may shorten the low-flow time to improve patient outcome [29, 36, 37].

Patient age is an important consideration when initiating E-CPR, taking into account the cost and benefit. Age is an important, but not the only, factor predicting survival after E-CPR for prolonged cardiac arrest [17]. In our study, compared with nonsurvivors, survivors had a higher proportion in the younger age group (< 55 years) (*p* = 0.018). In some studies, E-CPR use was excluded for patients over 60 years of age [4, 32]. We suggest that such younger-old patients should not be excluded from E-CPR use [38]. We consider 75 years of age an exclusion criterion for E-CPR use in our institution, similar to most other authors [17, 32, 38, 39], because of the low rate of benefits from E-CPR in such old-old patients.

Another important finding of our study is that nonshockable cardiac rhythm should not be the sole contraindication to E-CPR use for prolonged cardiac arrest. Many E-CPR programs exclude patients with nonshockable rhythms due to very limited outcomes after C-CPR [4, 40]. We found that compared with shockable cardiac arrest, nonshockable cardiac arrest had a worse prognosis. However, 10 of 49 patients (20.4%) presenting with nonshockable rhythms survived to discharge with E-CPR use. Our observations, along with those of other recent reports [3, 41, 42], suggest that we need to consider clinical parameters other than rhythm alone as contraindications to E-CPR use.

The current study is novel because it builds the TLR score (low-flow **T**ime, cardiac arrest **L**ocation, and initial cardiac arrest **R**hythm) with three pre-ECMO factors to stratify our patient outcome after receiving E-CPR with internal validation by bootstrap simulations. As previously mentioned, patient outcome with E-CPR is affected by a complex interplay of multiple factors [17, 21–23, 43]. Reported predictors of survival and neurological function include age, initial cardiac rhythm, low-flow time, arterial pH value, and serum lactate level [16, 21, 43]. These variable clinical predictors reveal not only the underlying patient characteristics but also the quality of the resuscitation process. For example, initial serum lactate is an indicator of tissue hypoxia [26, 44], but dynamic changes in lactate level also reflect the effect of ECMO therapy [16, 45]. In our study, EDCA was one factor in patient outcome, and such clinical parameters to predict patient outcome are important during E-CPR.

We identified a high-risk group of patients, i.e., those who had no favorable prognostic factors and thus a very low chance (0%) of benefit from E-CPR. Low-risk patients, i.e., those with > 14 points in our scoring system, were most likely to benefit from E-CPR following refractory cardiac arrest, with a 65.8% survival rate at discharge. E-CPR has the potential to put patients who are at the end of their natural life through a very invasive intervention with no meaningful likelihood of benefit. In addition, the cost of E-CPR is substantial [8], and we believe that such a risk stratification is helpful to the physician for identifying appropriate patients for E-CPR. However, a larger study involving more patients with external validation of our observations is required.

Our study has several limitations. First, the study was retrospective, and the number of cases was limited. Second, the quality of CPR performed by personnel in the prehospital setting, the in-hospital setting, and at the emergency department differed and was difficult to evaluate, and it may have been an important cofounding factor influencing patient outcome. Finally, the proposed risk stratification system is used to stratify patient outcome only in our institution, which is equipped to perform E-CPR even in the emergency department. Future prospective studies with larger sample sizes are required to verify these findings and extrapolate them to the wider population.

## Conclusions

This study has created a risk stratification system for predicting survival of E-CPR for refractory cardiac arrest. The score identifies a low-risk group of patients with a high rate of survival to discharge (65.8%) and good neurological function at discharge (60.5%) and a high-risk group with a zero rate of survival to discharge. Such risk stratification with pre-E-CPR parameters to predict patient survival after E-CPR may help clinical decision-making for E-CPR implementation.

## Abbreviations

C-CPR: Conventional cardiopulmonary resuscitation
CPC: Cerebral performance category
CPR: Cardiopulmonary resuscitation
E-CPR: Extracorporeal cardiopulmonary resuscitation
ECMO: Extracorporeal membrane oxygenation
EDCA: Emergency department cardiac arrest
IHCA: In-hospital cardiac arrest
OHCA: Out-of-hospital cardiac arrest
ROSC: Return of spontaneous circulation
SAVE: Survival After Veno-arterial ECMO
